# Spiritual well-being and associated factor among adult cancer patients in Hawassa University Comprehensive Specialized Hospital, Oncology Center, Hawassa, Ethiopia

**DOI:** 10.3389/fonc.2024.1357506

**Published:** 2024-05-01

**Authors:** Fekadu Abera Kebede, Bontu Hailu Tafesse, Fikre Moga, Addisalem Haile, Ebisa Zerihun

**Affiliations:** ^1^ Department of Nursing, College of Health Science, Oda Bultum University, Chiro, Ethiopia; ^2^ Faculty of Health Sciences, Hawassa University, Hawassa, Ethiopia; ^3^ College of Medicine and Health Sciences, Arba Minch University, Arba Minch, Ethiopia

**Keywords:** spiritual well-being, cancer, mental distress, oncology center, Hawassa, Ethiopia

## Abstract

**Background:**

Spiritual well-being has been shown to boost resistance to mental health crises in cancer patients during the diagnosis and treatment process, but there is a paucity of studies about spirituality in cancer patients, which may make it difficult for healthcare clinicians to assess spirituality and provide spiritual care.

**Objective:**

The aim of this study was to assess the level of spiritual well-being and associated factors among cancer patients in HUCSH Oncology Center in 2022.

**Methods and materials:**

An institution-based cross-sectional study was done from May 30 to June 30, 2022 among 267 cancer patients, and the respondents were selected by a simple random sampling technique. Data was collected by using standardized interviewer-administered questionnaires (FACIT sp12). Data was entered using Epi data version 4.6, and analysis was carried out by using Statistical Package for Social Science version 25. Bivariate and multivariate logistic regression was conducted to determine the relationship between the independent and dependent variables. The strength of association was tested by using *p*-value at 95% CI. Ethical clearance was obtained from the Institutional Review Board of Hawassa University College of Medicine and Health Science. During data collection, the purpose of the study was clearly explained to the patients, and consent was obtained.

**Result:**

A total of 267 cancer patients were included in the study. There was 100% response rate. Majority of the patients (80.5%) were in a poor spiritual well-being state. Mental distress (AOR = 0.246; 95% CI: 0.114–0.531) and religious education (AOR = 1.288; 95% CI: 1.438–9.142) were factors significantly associated with spiritual well-being among cancer patients.

**Conclusion and recommendation:**

This study showed that more than two-thirds of patients had poor spiritual well-being. Mental distress and religious education were factors associated with spiritual well-being. Attention should be given by nurses of the center for spiritual well-being assessment in clinical practices favoring holistic care in the center.

## Introduction

Cancer is a broad word for a disorder in which the body’s cells begin to multiply and expand uncontrollably, which can be caused by interactions between genetic and environmental factors producing aberrant alterations (1). Physical carcinogens (such as ionizing radiation), chemical carcinogens (such as asbestos, tobacco smoke components, and aflatoxins), and biological carcinogens (e.g., certain viruses, bacteria, and parasites) are all risk factors found in the environment (2–4). Cancer risk factors are strongly linked to socioeconomic position; they are stronger in low-socioeconomic-status groups, where cancer survival is lower than in wealthier social situations (5–7). Cancer imposes an enormous burden on society in low- and high-income countries (8). Because of population expansion and age, as well as an increase in the frequency of proven risk factors such as smoking, obesity, physical inactivity, and changing reproductive patterns linked with urbanization and economic development, cancer is becoming more common (9).

Spirituality is a sense of connection with people, having meaning and purpose in life, and, additionally, believing in and relating to a superior or higher force (10). Spiritual well-being and spiritual health are two ideas associated with spirituality. The meanings of these two notions themselves overlap but are separate. A person’s state of spiritual health can help them discover their life’s meaning and purpose as well as experience love, happiness, tranquility, and the beauty of nature (11–14).

People who have a strong sense of spiritual health feel connected to a higher power, to others, and to life in general. This is a powerful method to keep a good outlook in life despite any potential adverse situations that may arise (15–18). Those people also have a clear understanding of the meaning and purpose of life and are always reflecting on and working on improving themselves (11).

Religion and spirituality are two different things; the study showed that religion is a multifaceted construct that is focused on institutions and traditions. Additionally, it is defined by norms, rules, dogmas, and rituals. It unites people who have the same views. On the other hand, spirituality is viewed as a more intimate aspect, of a wider construct and one wherein an individual endeavors to uncover the sacred meaning of life without confessional restrictions (19–22).

The study showed that spirituality and religious beliefs are crucial, particularly for people who have serious illnesses or are dealing with life-threatening health issues (23). Patients with cancer initially experience shock or denial, followed by emotional trauma, anxiety, lack of concentration, difficulty falling asleep, loss of appetite, irritability, and intrusive worry about the future (24). Therefore, spirituality is a crucial component in giving cancer patients a context for finding meaning and hope in coping with their sickness from diagnosis through treatment, survival, recurrence, and death. It may also operate as a protector by acting as a buffer between the negative effects of illness and stress in daily life. Several studies revealed that spirituality has a tremendous role to cancer patients and may influence medical decision-making. Spiritual interventions should be taken into consideration and implemented into the care plan for each cancer patient because spirituality is a crucial component of holistic and person-centered care (23, 25–27).

In the upcoming 2030, the burden of cancer is predicted to increase globally, particularly in developing nations. Less developed countries currently account for about 57% of cancer diagnoses and 65% of cancer deaths globally as a result of the shifting global cancer burden (9). Despite the fact that the mortality rates in more industrialized nations are just 8% to 15% higher than those in less developed nations, more developed nations have cancer incidence rates that are twice as high (28). The overall burden of cancer in the world is projected to continue to rise, particularly in developing countries (29).

A study showed that spiritual or religious practices can assist patients to better manage the effects of cancer and its treatment (30). Increased optimism and hope are linked to spirituality as are regretness and a peaceful internal frame of mind (31). While there are numerous therapies to address the psychological effects of illness and enhance the quality of life of cancer patients, spiritual health has gained increased significance in studies on cancer survivorship (32–34).

A study revealed that low spiritual well-being has a tremendous association with suicidal thoughts, a wish to die, and hopelessness. Spiritual well-being is routinely ignored (35). The majority of studies on cancer patients’ well-being and quality of life revealed a favorable correlation between overall spiritual well-being and QOL, which was not equal across physical, social, emotional, and functional well-being (36).

Spiritual needs, which are frequently defined as spiritual challenges, spiritual yearning (e.g., asking forgiveness), and the desire for purpose and meaning in life post-diagnosis, affect approximately 86% to 91% of people with advanced cancer (37). For individuals with life-threatening illnesses, spirituality is especially crucial. Despite research demonstrating the advantages of spiritual assessment and care for terminally ill patients, practical practice rarely take their spiritual needs into account (38–40). There is a paucity of studies about spirituality in cancer patients, which may make it difficult for healthcare clinicians to assess spirituality and provide spiritual care. Therefore, the purpose of the current study is to assess the level of spiritual well-being and associated factors among cancer patients in HUCSH, Ethiopia.

## Materials and methods

### Study design and setting

An institution-based cross-sectional study design was conducted from May 30 to July 30, 2022 at Hawassa University Comprehensive Specialized Hospital. The study was conducted at Hawassa University Comprehensive Specialized Hospital Cancer Center, Hawassa, Ethiopia. Hawassa City is 275 km away from Addis Ababa. Hawassa University Comprehensive Specialized Hospital (HUCSH) is accompanied with various units such as dermatology clinic, laboratory unit, pathology unit, psychiatry clinic, ENT clinic, physiotherapy unit, surgical unit (general surgery, neurosurgery, orthopedics, urology, and plastic surgery), internal medicine with sub-specialty of cardiology and neurology, ophthalmology, radiology, oncology, gynecology and obstetrics, and pediatrics to serve the community.

### Study population

All cancer patients (survivors) with all kinds of cancer stages who were attending their follow-up in HUCSH Oncology Center in 2022 were included.

### Sample size determination and sampling technique

The sample size was determined by using the formula for estimating a single population proportion formula. A single proportion formula was used with 80.26% proportions (31), and after adding 10% response rate, the sample size was 267.


n=(Za2)2 (p)(q)W2n=(1.96)2 (0.8026)(0.1974)0.052243

where *Z* is the reliability coefficient for the desired confidence interval (*Z* for 95% is 1.96), *p* is the proportion (prevalence of spiritual well-being = 80.26%), *W* is the margin of sampling error tolerated (taking 5%), and *n* is the number of samples.

Upon adding 10% non-response rate, the total sample size was 267.

After obtaining ethical clearance and permission from Hawassa University Comprehensive Specialized Hospital, the study was conducted among cancer patients who came for initiation of treatment and for follow-up in HUCSH. The respondents were selected by using simple random sampling technique.

### Variables and measurement

The study’s dependent variable was spiritual well-being (good and poor). Socio-demographic characteristics, comorbidity, type of cancer, stage of cancer, mental distress, pain severity, and social support were the independent variables.

### Data collection instrument (tool) and procedure

All of the FACIT-Sp questionnaires were designed for an interview-based approach and used a 5-point Likert type scale to measure patient-reported HRQOL (0 = not at all, 1 = a little bit, 2 = somewhat, 3 = quite a bit, and 4 = very much). The recall period for each questionnaire is 30 min. The FACIT-Sp questionnaire contains a total of 12 questions with three main parts: (1) meaning of life (sp2, sp3, sp5, and sp8), (2) peace (sp1, sp4, sp6, and sp7), and (3) faith (sp9, sp10, sp11, and sp12). The FACIT-Sp validated and reliable tool had a validity of *α* = 0.81–0.88 and internal reliability of *α* = 0.9. The questionnaire was adopted from a study done in Addis Ababa (31, 41). Pain intensity was assessed using a four-point verbal rating scale (42). Mental distress was assessed by using the SRQ20 screening instrument which has 20 questions. Lastly, social support was assessed by using Oslo 3 (Emebet Girma, February, 2, 2015). The questionnaire was divided into the following four sections:

Section 1: Socio-demographic characteristics and clinical factor of the respondents.
Section 2: Spiritual well-being (FACIT-sp).
Section 3: Social support (Oslo 3).
Section 4: SRQ20 (to collect information on mental distress).

A face-to-face interview questionnaire was administered among cancer patients in the selected study areas, and clinical information were collected from the patients’ files. All cancer patients who fulfill the inclusion criteria were interviewed. Two supervisors and four data collectors were recruited among nurses in the center.

### Data quality control measures

To keep the quality of the data, 5% of the questionnaires (14 questionnaires) were pre-tested at Alatyon Primary Hospital on some patients a week before the actual data collection period. The vague terms, phrases, and questions identified during the pre-test were modified and changed. The investigator conducted an orientation for 2 days with the data collectors on how to collect data regarding all variables on the questionnaire to keep the integrity and quality of the data. The investigator and supervisors monitored the data collectors during the time of the data collection process by cross-checking the data for completeness and consistency of the gathered information.

### Data processing and analysis

Data was entered into EPI data version 4.6 software and exported to SPSS version 26 for data analysis. Descriptive statistics such as frequency and percentages were obtained to summarize the data. Bivariate and multivariate analyses were carried out to examine the relationship between the outcome variable and the predictor variables. Variables that have a *p*-value less than 0.25 upon bivariate analysis were entered into the multivariable logistic regression. Adjusted odds ratios (AOR) and their 95% CI were used as indicators of the strength of association. Statistical significance was set at *p*-value of less than 0.05 on multivariate analysis. The result was described in sentences and displayed in tables, graphs, and charts. Hosmer and Lemeshows fitness model was used to check the model fitness. Multicollinearity was checked by using variance inflation factor and tolerance.

### Operational definitions

#### Good spiritual well-being

The total score is the sum of the scores of the subscales, which range from 0 to 48. Good SWB was defined as a FACIT-Sp total score ≥36.

Poor spiritual well-being was defined as a FACIT-Sp total score<36 (31).

#### Mental distress

Each of the 20 items scores 0 or 1. A score of 1 indicates that the symptom was present in the past month, whereas 0 indicates that the symptom is absent and is defined as a total score ≥8.

No mental distress was defined as a total score<8.

#### Poor social support

The total score ranges from 3 to 14 and defined as a score ranging from 3 to 8 (Emebet Girma, February 2, 2015).

Moderate social support: defined as a score that ranges from 9 to 11.

Strong social support: defined as a score that ranges from 12 to 14.

## Results

### Socio-demographic of cancer patients

A total of 267 participants were included in this study, and there was 100% response rate. Age was in the range of 18–80 years old, and most participants were in the age group of 31–45 years old 33.3% (*n* = 89). Most of the participants were women (56.2%, *n* = 150), and 35.6% (*n* = 95) were Muslim in religion. More than half of the patients 65.5% (*n* = 175) were married. Regarding level of education and religious education, 34.9% (*n* = 93) attended secondary school and 76% (*n* = 203) have not taken religious education. More than half of the patients 58.4% (*n* = 156) were rural residents, and 37.1% (*n* = 99) of the respondents had an income less than 1,000 ETB. According to the results, most participants 87.3% (*n* = 233) had no religious responsibility (**Table 1**). 

**Table 1:** Distribution of the socio-demographic variables of patients in HUCSH Ethiopia, 2022 (n = 267).

Variables	Frequency	Frequency (%)
Sex		
Male	117	43.8
Female	150	56.2
Age		
18-30	55	20.6
31-45	89	33.3
46-60	79	29.6
61-75	40	15
>75	4	1.5
Marital status		
Single	47	17.6
Married	175	65.5
Divorced	27	10.1
Widow	18	6.7
Religion		
Muslim	95	35.6
Orthodox	68	25.5
Protestant	64	24
Catholic	23	8.6
Other	17	6.4
Residence		
Rural	156	58.4
Urban	111	41.6
Income in Birr		
<1000	99	37.1
1001-3000	71	26.6
3001-5000	67	25.1
5001-7500	22	8.2
>7500	8	3
Educational status		
Can’t read & write	61	22.8
Read and write	42	15.7
Primary school	66	24.7
Secondary school	93	34.9
College diploma and above	5	1.9
Religious education		
Yes	64	24
No	203	76
Religious responsibility		
Yes	34	12.7
No	233	87.3
Social support		
Poor	81	30.3
Moderate	95	35.6
Strong	91	34.1
Variables	Frequency	Frequency (%)
Sex		
Male	117	43.8
Female	150	56.2
Age		
18–30	55	20.6
31–45	89	33.3
46–60	79	29.6
61–75	40	15
>75	4	1.5
Marital status		
Single	47	17.6
Married	175	65.5
Divorced	27	10.1
Widow	18	6.7
Religion		
Muslim	95	35.6
Orthodox	68	25.5
Protestant	64	24
Catholic	23	8.6
Other	17	6.4
Residence		
Rural	156	58.4
Urban	111	41.6
Income (birr)		
<1,000	99	37.1
1,001–3,000	71	26.6
3,001–5,000	67	25.1
5,001–7,500	22	8.2
>7,500	8	3
Educational status		
Cannot read and write	61	22.8
Read and write	42	15.7
Primary school	66	24.7
Secondary school	93	34.9
College diploma and above	5	1.9
Religious education		
Yes	64	24
No	203	76
Religious responsibility		
Yes	34	12.7
No	233	87.3
Social support		
Poor	81	30.3
Moderate	95	35.6
Strong	91	34.1

### Clinical characteristics of cancer patients

Breast cancer (25.1%, *n* = 67) was the most common cancer type, and 82.4% (*n* = 220) of the respondents had a comorbidity. Moreover, 44.6% (*n* = 119) of the respondents are in stage 3 of cancer, half of the respondents (50.6%, *n* = 135) were in moderate pain, and more than one-third (35.6%, *n* = 95) of the respondents had moderate social support. Lastly, more than half of the respondents had mental distress (55.8%, *n* = 149) (**Tables 2**, **3**).

**Table 2:** Psychological variables.

Variable	Frequency	Frequency percentage
Mental health		
No mental distress	118	44.2
Mental distress	149	55.8

**Table 3:** Frequence of clinical variables.

Variables	Frequency	Frequency Percentage
Pain severity		
Mild	36	13.5
Moderate	135	50.6
Severe	72	27
Very severe	24	9
Comorbidity		
Yes	47	17.6
No	220	82.4
Stage of cancer		
Stage 1	21	7.9
Stage 2	22	8.2
Stage 3	119	44.6
Stage 4	105	39.3

### Prevalence of spiritual well-being among adult cancer patients in HUCSH

**Figure 1:**
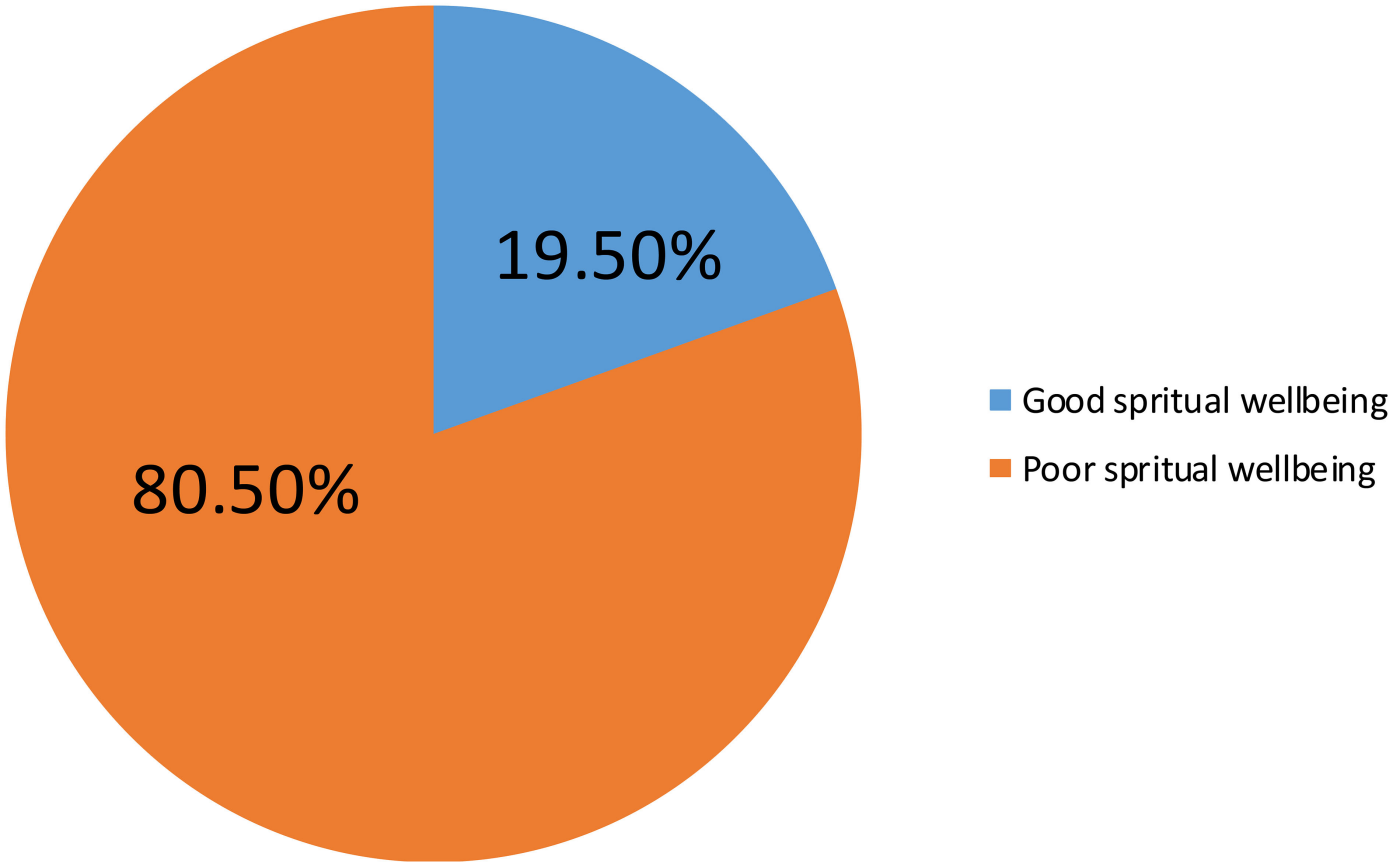
Prevalence of spiritual well-being and associated factors among cancer patients in HUCSH, Ethiopia, 2022 (267).

In current study, more than three quarters (80.50%) of participants had poor spiritual wellbeing, whereas nearly a quarter (19.50%) of participants had good spiritual wellbeing (**Figure 1**).

#### Factors associated with spiritual well-being

In the binary logistic regression model, religion, religious education, religious responsibility, and mental distress were significantly associated with the spiritual well-being of cancer patients, with a *p*-value of less than 0.025 (**Table 4**).

**Table 4:** Result of binary logistic regression of variables’ association with spiritual well-being among cancer patients in Oncology Center at HUCSH 2022 (n = 267).

Variable	Spiritual category		COR	*P*-value	CI	
	Good	Poor			Upper	Lower
Religion						
Protestant	9	55	3.333	0.006	1.407	7.898
Muslim	9	86	5.212	0.000	2.233	12.168
Catholic	7	16	1.247	0.671	0.45	3.456
Adventist	3	14	2.544	1.73	0.665	9.745
Orthodox	24	44				
Religious education						
No	28	175	3.75	0.000	1.969	7.143
Yes	24	40				
Religious responsibility						
Yes	12	22	2.632	0.015	1.205	5.72
No	40	193				
Mental distress						
Mental distress	17	132	3.274	0.000	1.724	6.217
Mental distress	35	83				

#### Factors associated with spiritual well-being among cancer patients at HUCSH

In multiple logistic regression models, religious education and mental distress were significantly associated with the spiritual well-being of a cancer patient, with a *p*-value less than 0.05. However, religion of respondents and religious responsibility were not significantly associated with the spiritual well-being of a cancer patient (**Table 4**). Compared to patients who take religious education, patients who did not take religious education were three times more likely to have poor spiritual well-being (AOR = 3.8; 95% CI: 1.573–9.342). In relation to patients with no mental distress, patients with mental distress were four times more likely to have poor spiritual well−being (AOR = 4.3; 95% CI: 2.103–8.982) (**Table 5**).

**Table 5:** Result of multiple logistic regression of variables’ association with spiritual well-being among cancer patients in Oncology Center at HUCSH 2022 (n = 267).

Variables	AOR	Lower CI	Higher CI	*P*-value
Mental health	4.347	2.103	8.982	0.000
Protestant	2.654	0.639	11.02	0.179
Muslim	0.778	0.174	3.489	0.743
Catholic	0.462	0.102	2.081	0.314
Adventist	2.137	0.41	11.13	0.367
Religious education	3.833	1.573	9.342	0.003
Religious responsibility	1.056	0.352	3.161	0.923

## Discussion

Cancer is a life-threatening issue, and patients faced stress, which affects the patients’ pre- and post-treatment as well as overall health issues. Patients viewed spirituality as a healthy source of power and recovery. This research was undertaken to investigate the proportion of spiritual well-being among cancer patients of HUCSH Oncology Center. The final finding of our study showed a high prevalence of poor spiritual well-being (80.5%, *n* = 215). This result is almost similar to that of a study done in Addis Ababa (80.26%, *n* = 347), Indonesia (84.9%, *n* = 51), and Brazil (76%, *n* = 69) (15, 27, 21). In contrast to this, the prevalence of poor spiritual well-being was even low when compared with some studies done in different countries like Pakistan (29.5%, *n* = 59) and Portugal (64%, *n* = 96) (22,21). The possible explanation for this variation might be methodological differences like geographical (study) area, different assessment tool, number of samples, and assessment time gap—for instance, a study done in Portugal shows 150 samples, and the tool they had used was SWBQ with 20 items, but our study used FACIT-SP12 with 12 items. On the other hand, the difference may be due to a knowledge gap or a negative and unaware attitude toward the nature of the disease process (cancer).

After fitting a multiple model and considering confounding variables, the variables that remain associated with spiritual well-being were mental distress and religious education.

In this study, mental distress was significantly associated with spiritual well-being (AOR = 4.347; 95% CI: 2.103–8.982). In relation to patients with no mental distress, patients with mental distress were four times more likely to have poor spiritual well−being, and this finding is in line with the finding obtained from Italy (30). Religious education is significantly associated with spiritual well-being. Cancer patients who did not take religious education were three times higher in having poor spirituality (AOR = 3.833, *P* = 0.003) when compared with patients who have taken religious education. This result is consistent with the result obtained from a study done in Ethiopia Addis Ababa Tikur Anbesa Hospital in 2021 (31). This finding might be explained by the fact that many cancer patients saw their recovery from their cancer as an opportunity to grow closer to God the more religiously educated they were.

### Strengths and limitations

#### Strengths

➢ An adequate sample was taken from the reference population.

➢ The study identified factors associated with the spiritual well-being of the participants.

#### Limitations

✥ Few studies had assessed the role of SWB and its association with a diverse set of variables in this population, which has limited the scope of the discussion (lack of materials on related topics).

✥ There was a short time frame.

## Conclusion

A total of 267 participants were included in this study, and there was 100% response rate. From the result of this study, the researcher revealed that two-thirds of the respondents reported poor spiritual well-being in Hawassa University Specialized Hospital Oncology Center in 2022. Among variables, religious education and mental health were significantly associated with spiritual well-being. Based on the findings of the study, the recommendations were forwarded to the concerned bodies, particularly Hawassa University Comprehensive Specialized Hospital, religious institutions, and researchers.

## Data availability statement

The original contributions presented in the study are included in the article/supplementary material. Further inquiries can be directed to the corresponding author.

## Ethics statement

The studies involving humans were approved by Institutional Review Bored of Hawassa University College of Medicine and Health Science. The studies were conducted in accordance with the local legislation and institutional requirements. The participants provided their written informed consent to participate in this study. Written informed consent was obtained from the individual(s) for the publication of any potentially identifiable images or data included in this article.

## Author contributions

FK: Conceptualization, Data curation, Formal analysis, Investigation, Methodology, Project administration, Resources, Software, Supervision, Validation, Visualization, Writing – original draft, Writing – review & editing. BT: Conceptualization, Data curation, Formal analysis, Investigation, Methodology, Project administration, Resources, Software, Supervision, Validation, Visualization, Writing – original draft, Writing – review & editing. FM: Visualization, Writing – original draft, Writing – review & editing, Conceptualization, Data curation, Formal analysis, Investigation, Methodology, Project administration, Resources, Software, Supervision, Validation. AH: Conceptualization, Data curation, Formal analysis, Investigation, Methodology, Project administration, Resources, Software, Supervision, Validation, Visualization, Writing – original draft, Writing – review & editing. EZ: Conceptualization, Data curation, Formal analysis, Investigation, Methodology, Project administration, Resources, Software, Supervision, Validation, Visualization, Writing – original draft, Writing – review & editing.
